# L-methionine supplementation modulates IgM^+^ B cell responses in rainbow trout

**DOI:** 10.3389/fimmu.2023.1264228

**Published:** 2023-10-10

**Authors:** Diana Martín, M. Camino Ordás, Inês Carvalho, Patricia Díaz-Rosales, Noelia Nuñez-Ortiz, Samuel Vicente-Gil, Aitor Arrogante, Carlos Zarza, Marina Machado, Benjamín Costas, Carolina Tafalla

**Affiliations:** ^1^ Fish Immunology and Pathology Laboratory, Animal Health and Research Center (CISA), National Institute for Agricultural and Food Research and Technology (INIA), Spanish National Research Council (CSIC), Madrid, Spain; ^2^ Centro Interdisciplinar de Investigação Marinha e Ambiental (CIIMAR), Universidade do Porto, Matosinhos, Portugal; ^3^ Instituto de Ciências Biomédicas Abel Salazar (ICBAS), Universidade do Porto, Porto, Portugal; ^4^ Skretting Aquaculture Innovation, Stavanger, Norway

**Keywords:** amino acids, methionine, tryptophan, lysine, IgM ^+^ B cells, rainbow trout, TNP-LPS

## Abstract

The interest in dietary amino acids (AAs) as potential immunomodulators has been growing the recent years, since specific AAs are known to regulate key metabolic pathways of the immune response or increase the synthesis of some immune-related proteins. Methionine, tryptophan and lysine are among the ten essential AAs for fish, meaning that they cannot be produced endogenously and must be provided through the diet. To date, although dietary supplementation of fish with some of these AAs has been shown to have positive effects on some innate immune parameters and disease resistance, the effects that these AAs provoke on cells of the adaptive immune system remained unexplored. Hence, in the current study, we have investigated the effects of these three AAs on the functionality of rainbow trout (*Oncorhynchus mykiss*) IgM^+^ B cells. For this, splenic leukocytes were isolated from untreated adult rainbow trout and incubated in culture media additionally supplemented with different doses of methionine, tryptophan or lysine in the presence or absence of the model antigen TNP-LPS (2,4,6-trinitrophenyl hapten conjugated to lipopolysaccharide). The survival, IgM secreting capacity and proliferation of IgM^+^ B cells was then studied. In the case of methionine, the phagocytic capacity of IgM^+^ B cells was also determined. Our results demonstrate that methionine supplementation significantly increases the proliferative effects provoked by TNP-LPS and also up-regulates the number of cells secreting IgM, whereas tryptophan or lysine have either minor or even negative effects on rainbow trout IgM^+^ B cells. This increase in the number of IgM-secreting cells in response to methionine surplus was further verified in a feeding experiment, in which the beneficial effects of methionine on the specific response to anal immunization were also confirmed. The results presented demonstrate the beneficial effects of dietary supplementation with methionine on the adaptive immune responses of fish.

## 1 Introduction

Nutrition has been shown to have significant implications on animal health. Particularly, in fish, best practices on diet formulation are of major importance, as feeds usually represent the leading expenditure to the aquaculture industry (1). In fact, the incorporation of functional ingredients in aquafeeds is currently recognized as an effective farming tool to significantly improve fish robustness. The term functional or fortified feed is used to describe feeds that have added benefits beyond the fish essential nutritional requirements, improving both the immunological status and growth (2, 3). Hence, a large number of additives or feed ingredients are becoming available for inclusion in functional feeds such as prebiotics, probiotics, β-glucans or nucleotides (1, 4), allowing a reduction of chemotherapeutic and antibiotic treatments. Among the potential feed additives that can regulate the immune status of fish, to date, little attention has been paid to individual amino acids (AAs). However, already in 2010, Wu proposed a new concept of functional AAs, defined as those AAs that participate in and regulate key metabolic pathways to improve, for instance, health and survival (5). Since then, the past recent years have witnessed a growing interest in the biochemistry of AAs and their effects in growth, health and disease in humans and other animal models (6). Additionally, AA requirements may increase as a direct consequence of metabolic changes associated with inflammation and infection (7). Hence, the dependency of the immune system on the availability of some specific AAs is associated with their role as signaling molecules essential for cellular functions of immune cells (3). For instance, AAs have been shown in higher vertebrates to be able to regulate the activation of T cells, B cells, natural-killer (NK) cells and macrophages by conditioning the cellular redox stage and cytokine secretion and production of radicals such as nitric oxide (NO) and superoxide (reviewed in (8, 9)).

Methionine and tryptophan are two examples of indispensable AAs (IAAs) with recognized roles in the immune system and their dietary supplementation has proved to enhance mammalian host immunity (9). Through the generation of decarboxylated S-adenosylmethionine, methionine is a donor of the methyl group that participates in the methylation of DNA and proteins, the synthesis of the polyamines spermidine and spermine, and in the regulation of gene expression (2). Methionine also plays a pivotal role in processes responsible for the control of inflammation and apoptosis such as ubiquitination of proteins (10) and autophagy (11) by inducing S-adenosylmethionine methylation. Methionine dietary immunomodulation has also been explored in modern animal production. For instance, the use of methionine as a nutraceutical supplement has been proposed to control enteric processes and oxidative stress in mammals (12). Similarly, the beneficial effects of dietary supplementation with methionine (and other sulfur-containing AAs) on poultry immune mechanisms and its use on poultry industry have also been widely explored (13, 14). In fish, methionine supplementation was shown to modulate the immune response of different fish species to an inflammatory insult as well as to increase disease resistance (15–17).

Tryptophan is another IAA with important roles in protein synthesis that acts as precursor of several compounds with a wide range of effects in the modulation of the stress response, the antioxidant system, as well as behavioral and immune responses (18). In contrast, inflammatory cytokines released during immune stimulation activate indoleamine 2,3-dioxygenase (IDO), which catabolizes tryptophan leading to its depletion through the kynurenine pathway (19). Interestingly, depletion of plasma tryptophan or increased kynurenine–tryptophan ratio have been reported during viral, bacterial or intracellular parasitic infections (20). Given the progressive decline of tryptophan in the plasma of animals with inflammation, its catabolism plays a critical role in the functions of both macrophages and lymphocytes (21). However, the potential use of tryptophan supplementation in animal health management has not been widely explored. In fish, the dietary supplementation with tryptophan seemed as a promising strategy to overcome disease susceptibility in situations of chronic stress (22), yet its potential as an immunomodulator still needs to be evidenced (17).

Lysine is another IAA that plays a crucial role in health and growth in a wide variety of vertebrate animals (3). In higher vertebrates, lysine restriction has been shown to affect inflammatory responses and acquired immunity. For instance, lysine deficiency impaired inflammatory cytokine production, antibody responses and cell-mediated immune responses in piglets (23) and broilers (24). In fish, some studies have reported benefits of dietary lysine surplus on growth performance (3). However, the role of lysine on the immune function of fish has received limited attention to date.

In this context, in the current study, we have investigated the role of methionine, tryptophan and lysine supplementation on IgM^+^ B cells of rainbow trout (*Oncorhynchus mykiss*) through a series of *in vitro* experiments. For this, splenic leukocytes were isolated and cultured in regular medium or in medium supplemented with different concentrations of each of the three IAAs, to then determine the response of IgM^+^ B cells to a model antigen, 2,4,6-trinitrophenyl hapten conjugated to lipopolysaccharide (TNP-LPS). We have assessed the survival, proliferation and IgM secretion of these IgM^+^ B cells. In the case of methionine, we have also established its effects on the phagocytic capacity of IgM^+^ B cells. Finally, a feeding experiment was undertaken with three diets containing different amounts of methionine, to confirm some of the results obtained *in vitro*. These fish were also immunized with TNP-LPS anally and the specific antibody response measured after 1 month. Our results demonstrate that methionine supplementation has the most beneficial effects on B cell function in teleost fish, and points to this IAA as an adequate supplement to improve acquired immune defenses in farmed fish.

## 2 Materials and methods

### 2.1 Fish

Rainbow trout (*Oncorhynchus mykiss*) of approximately 100 g obtained from Piscifactoria Cifuentes (Cifuentes, Guadalajara, Spain) were used to obtain splenic leukocyte populations and perform the *in vitro* analyses. These fish were maintained at the animal facilities of the Animal Health Research Centre (CISA-INIA-CSIC, Spain) in an aerated recirculating water system at 16°C, with a 12:12 h light: dark photoperiod. Fish were fed twice a day with a commercial diet (Skretting). Prior to sampling, fish were acclimatized to laboratory conditions for at least 2 weeks. During this period, no clinical signs of disease were ever observed.

Rainbow trout of approximately 35 g obtained from Piscifactoria Riomundo (Albacete, Spain) were used in the feeding experiment. These fish were maintained at Aquaculture Research Center (ITACyL, Segovia, Spain) in the same conditions as described above. Prior to starting the feeding trial, fish were acclimatized to laboratory conditions for 4 weeks. During this period, all fish were fed the control diet later used in the experiments and no clinical signs of disease were observed.

All the experiments described comply with the Guidelines of the European Union Council (2010/63/EU) for the use of laboratory animals and have been approved by the INIA Ethics Committee (PROEX 065.3/21).

### 2.2 Leukocyte isolation

Rainbow trout were euthanized by benzocaine (Sigma-Aldrich) overdose and spleen was removed to isolate the total leukocyte populations. Cell suspensions were obtained passing the spleens through 100 μm nylon cell strainers (BD Biosciences) using Leibovitz’s medium (L-15, Gibco) containing 100 I.U./ml penicillin and 100 μg/ml streptomycin (P/S, Life Technologies), 10 I.U./ml heparin (Sigma-Aldrich) and 2% fetal calf serum (FCS, Thermo Fisher Scientific). Cell suspensions were then placed onto 30/51% Percoll (GE Healthcare) density gradients and centrifuged at 500 x *g* for 30 min at 4°C, without brake. The interface cells were collected and washed with L-15 supplemented with antibiotics and 2% FCS. The viable cell concentration was determined by trypan blue (Sigma-Aldrich) exclusion and cells were resuspended in RPMI medium (Gibco) supplemented with P/S and 5% FCS at a concentration of 2x10^6^ cells/ml.

### 2.3 Supplementation of media with amino acids

To evaluate the effect of the L-methionine, L-tryptophan and L-lysine on the splenic B cell populations, different concentrations of these IAAs (0, 0.5, 1 and 1.5 mM) were added to total splenic leukocytes incubated in RPMI medium containing P/S and 5% FCS, immediately after their isolation. These concentrations were chosen based on previous *in vitro* studies performed in European sea bass (*Dicentrarchus labrax*) (17). L-methionine and L-tryptophan were acquired from Sigma whereas L-lysine was provided by ThermoFisher. Cells in all conditions (supplemented or not with the different amino acids) were either stimulated with 5 μg/ml of 2,4,6-trinitrophenyl hapten conjugated to lipopolysaccharide (TNP-LPS) or left unstimulated. The TNP-LPS was also added when required immediately after isolation. After 72 h of incubation at 20°C, the percentage of IgM^+^ B cells in cultures and their proliferative response were estimated by flow cytometry. The number of IgM-secreting cells was also estimated at this point by ELISpot. The phagocytic capacity of IgM^+^ B cells was also established by flow cytometry in the case of cultures supplemented or not with methionine.

### 2.4 Flow cytometry

Leukocytes were stained with anti-trout IgM [1.14 mAb mouse IgG1 coupled to R-phycoerythrin (R-PE), 1 µg/ml] (25) for 1 h at 4°C in the dark in staining buffer (L-15 without phenol red containing 2% FCS). After this time, cells were washed twice with staining buffer. The cell viability was checked by addition of 4’,6-diamine-2’-phenylindole dihydrochloride (DAPI 0.2 μg/ml). Cells were analyzed on a FACS Celesta™ flow cytometer equipped with BD FACSDiva software. Flow cytometry analysis was performed with FlowJo^®^ V10 (TreeStar).

### 2.5 B cell proliferation

The Click-IT^®^ EdU Alexa Fluor^®^ 488 Flow Cytometry Assay Kit (Life Technologies) was used to measure the proliferation of IgM^+^ B cells following manufacturer’s instructions. For this, 1 µM EdU (5-ethynyl-2′-deoxyuridine) was added to splenocytes exposed to TNP-LPS or not in media supplemented or not with the different amino acids after the 72 h incubation period. The cells were then incubated for an additional 24 h. At this point, the viability of the cells was determined using the LIVE/DEAD™ Fixable Near-IR Dead Cell Stain kit (Invitrogen), following the kit´s specifications. Then, cells were washed and stained with anti-IgM (1.14) coupled to R-PE (1 µg/ml) for 30 min at 4°C. Cells were then fixed, permeabilized, and incubated with specific reagents to detect the incorporation of EdU to the DNA of proliferating cells following the manufacturer’s instructions. Samples were then analyzed by flow cytometry as described above. The percentage of IgM^+^ B cells with incorporated EdU (proliferating IgM^+^ B cells) among the total IgM^+^ B cell population was calculated in each case.

### 2.6 ELISpot

ELISpot was used to quantify the number of total IgM-secreting B cells as previously described (26). After the 72 h incubation period, splenocytes exposed to TNP-LPS or not in media supplemented or not with the different amino acids were transferred to pre-coated ELISpot plates. For this, ELISpot plates containing Inmobilon-P membranes (Millipore) were activated with 70% ethanol for 30 s, coated with anti-trout IgM mAb (clone 1.14) at 2 μg/ml in PBS, and incubated overnight at 4°C in agitation. To block non-specific binding to the membrane, plates were then incubated with 2% BSA (bovine serum albumin) in PBS (phosphate buffer saline) for 2 h at room temperature (RT). At this point, leukocytes were added to the wells in duplicate and incubated for 24 h at 20°C. Cells were washed away five times with PBS and plates were blocked again with 2% BSA in PBS for 1 h at RT. After blocking, biotinylated anti-trout IgM mAb (clone 1.14) was added to the plates at 1 μg/ml and incubated for 1 h at RT. Following additional washing steps (five times in PBS), the plates were developed using streptavidin-HRP (Thermo Fisher Scientific) (100 ng/ml) for 1 h at RT, washed again with PBS and incubated with 3-amino 9-ethylcarbazole (Sigma-Aldrich) for 30 min at RT in the dark. The substrate reaction was stopped by washing the plates with tap water. Once the membranes were dried, the number of spots in each well was determined using an AID iSpot Reader System (Autoimmun Diagnostika GMBH).

### 2.7 Phagocytic activity

After 72 h of incubation, splenocytes exposed to TNP-LPS or not in media supplemented or not with methionine were collected and resuspended in L-15 medium without serum. The cells were then incubated with fluorescent beads (FluoSpheres R Microspheres, 1.0 μm, Crimson Red Fluorescent 625/645, 2% solids; Thermo Fisher Scientific) at a cell:bead ratio of 1:10 as described before for 3 h at 20°C (26). After the incubation period, cells were harvested by gently pipetting, and non-ingested beads were removed by centrifugation (100 x *g* for 10 min at 4°C) over a cushion of 3% (weight/volume) BSA (Fraction V; Fisher Scientific) in PBS supplemented with 4.5% (weight/volume) D-glucose (Sigma). Cells were then resuspended in staining buffer, labeled with anti-IgM-FITC (1.14) (1 μg/ml) for 1 h at 4°C in the dark in staining buffer. After this time, cells were washed twice with staining buffer. The cell viability was checked by addition of 4’,6-diamine-2’-phenylindole dihydrochloride (DAPI 0.2 μg/ml). Cells were analyzed on a FACS Celesta™ flow cytometer equipped with BD FACSDiva software. Flow cytometry analysis was performed with FlowJo^®^ V10.

### 2.8 *In vivo* feeding experiment

To confirm the positive effects provoked by methionine supplementation on B cells, a feeding experiment was performed. For this, three diets were formulated and manufactured by Skretting Aquaculture Innovation (Norway). The control diet was formulated with the ideal AA profile estimated for rainbow trout and was identical to the one used to feed the rainbow trout used to obtain splenocytes for the *in vitro* analysis. The two other diets (MET 1 and MET 2) were formulated to be identical to the control diet but supplemented with L-methionine at 0.57 or 0.98% of feed weight respectively, at the expenses of wheat gluten and wheat meal. After AA analysis the percentage of methionine was 0.84% for the control diet (2.11% of all AA) and 1.21 (3.11% of all AA) and 1.58% (3.93% of all AA) for MET 1 and MET 2, respectively, presenting these diets 44% and 88.1% more methionine than the control diet.

To undertake the feeding experiment, fish were randomly assigned to three identical tanks (16 fish per group) and received either the control or the methionine-supplemented diets (MET 1 or MET 2). The fish were fed twice a day with each corresponding diet during one month at a rate of 2% body weight per day. Thereafter, six fish from each group were sacrificed by benzocaine overdose. Blood was extracted with a heparinized needle from the caudal vein and diluted 10 times with L-15 medium supplemented with antibiotics, 5% FCS and 10 IU/ml heparin. Blood cell suspensions were placed onto 51% Percoll cushions, centrifuged at 500 x *g* for 30 min at 4°C. The interface containing total leukocytes were collected. The spleen was also removed from these fish to isolate the total leukocyte populations as described above. Blood and spleen leukocytes were resuspended in L-15 medium supplemented with P/S and 5% FCS at a concentration of 2x10^6^ cells/ml. Leukocytes were immediately transferred to ELISpot plates pre-coated with anti-trout IgM and the number of total IgM-secreting B cells was then quantified as described before.

The remaining 10 fish from each experimental group were anally immunized with 50 μg of TNP-LPS in 100 μl of saline solution (0.9% NaCl). The solution was introduced with a pippete tip through the anus, and into the posterior segment of the gut as previously described (27). Fish were fed for an additional month with the corresponding diets (Control, MET 1 or MET 2), and then sacrificed and blood removed from the caudal vein. The blood was let to clot at 4°C overnight and serum extraction was then performed by centrifugation at 4000 x *g* for 10 min at 4°C. Supernatants were collected and centrifuged again at 10000 x *g* for 10 min at 4°C. Serum was stored at -80°C until use.

### 2.9 ELISA

The presence of TNP-specific IgM in serum was estimated by ELISA as previously described (27). For this, microtitre plates were coated with 5 μg/ml of TNP-BSA (Biosearch technologies) in a volume of 100 μl PBS overnight at 4°C. Thereafter, non-specific binding sites were blocked by incubation with 1% BSA in PBS with 0.05% Tween 20 (PBT) for 1 h at RT. Plates were then washed with PBT and a 1/500 dilution of each serum sample in PBS 1% BSA added to each well and incubated for 1 h at RT. Serum samples from all groups were analyzed in duplicate wells. After washing three times with PBT, each well was incubated with 1 μg/ml biotinyilated anti-trout IgM mAb (clone 4C10) diluted in PBS 1% BSA for 1 h at RT. The plates were washed again three times in PBT and 100 ng/ml of HRP-streptavidin (Thermo Fisher Scientific) added to each well in 100 μl PBS 1% BSA. After incubation at RT for 1 h, 100 μl of o-phenylenediamine dihydrochloride substrate reagent (Sigma-Aldrich) were added to each well. The reaction was stopped after 15 min by adding 50 μl of 2.5 M H_2_SO_4_. Absorbances were recorded at 490 nm using a FLUOstar Omega (BMG Labtech) plate reader. Internal positive and negative control samples were also included.

### 2.10 Statistical analysis

Data handling, statistical analyses and graphic representation were performed using GraphPad Prism version 8 (GraphPad Software). Statistical analyses were performed by two-way analysis of variance (ANOVA). The means were compared by Tukey multiple-range test two-way. The differences between the mean values were considered significant on different degrees, where * means *p* ≤ 0.05, ** means *p* ≤ 0.01, *** means *p* ≤ 0.001.

## 3 Results

### 3.1 Effect of L-methionine, L-tryptophan and L-lysine supplementation on the survival of IgM^+^ B cells

**Figure 1:**
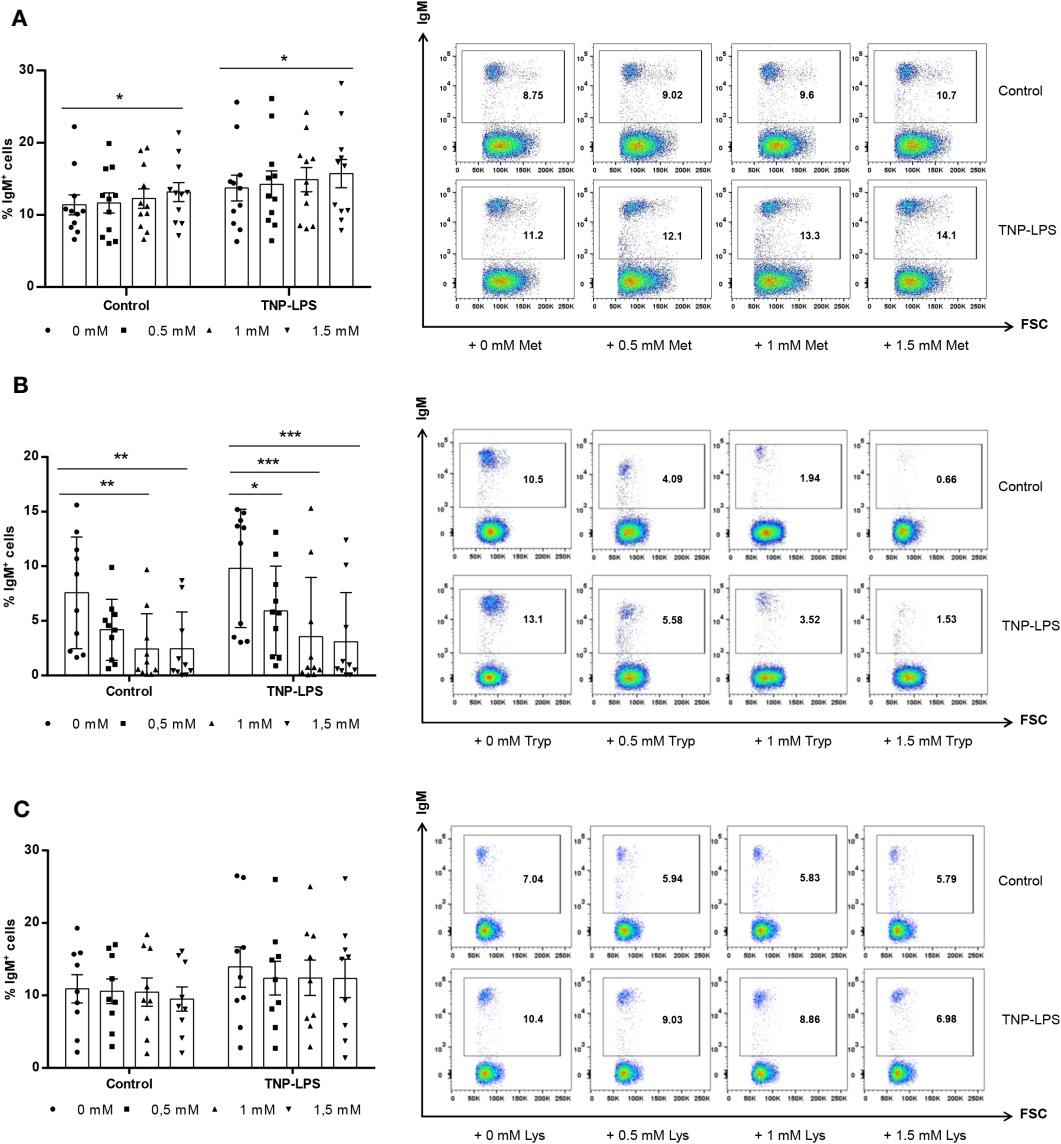
Effect of L-methionine, L-tryptophan and L-lysine supplementation on survival of IgM^+^ B cells. Spleen leukocytes were cultured in RPMI medium with 5% FCS and antibiotics supplemented with different concentrations (0.5, 1 and 1.5 mM) of L-methionine **(A)**, L-tryptophan **(B)** or L-lysine **(C)**. Non-supplemented controls incubated with RPMI media alone were also included. Some of these cultures were additionally stimulated with 5 μg/ml of TNP-LPS, while others were left unstimulated. All cells were incubated for 3 days at 20°C. At that point, the percentage of IgM^+^ B cells was determined through flow cytometry. Representative dot plots plots are shown along with graphs presenting the percentages of IgM^+^ B cells in splenocyte cultures (mean + SEM; n=11). Symbols represent individual values. Asterisks denote significantly different values between cultures supplemented with amino acids and non-supplemented controls as indicated (* means *p* ≤ 0.05, ** means *p* ≤ 0.01 and *** means *p* ≤ 0.001).

We studied whether supplementation of culture media with additional amounts of different amino acids affected the survival of IgM^+^ B cells in splenic leukocyte cultures. For this, different concentrations of L-methionine, L-tryptophan or L-lysine were added to rainbow trout splenic leukocyte cultures in RMPI media containing antibiotics and 5% FCS. Some of these cultures were stimulated with TNP-LPS, a model thymus independent (TI) antigen known to strongly stimulate rainbow trout B cells (28). After 72 h of incubation at 20°C, the percentage of IgM^+^ cells in cultures was established by flow cytometry. As shown in **Figure 1A**, when leukocyte cultures were supplemented with 1.5 mM L-methionine, the percentage of IgM^+^ B cells significantly increased in cultures. In the presence of TNP-LPS, the percentage of IgM^+^ B cells in cultures significantly increased when compared to non-stimulated cells, as previously described (28), being this increase significantly higher when the cultures were supplemented with 1.5 mM L-methionine (**Figure 1A**).

In contrast, the supplementation of leukocyte cultures with 1 mM or 1.5 mM L-tryptophan significantly decreased the percentage of IgM^+^ B cells in non-stimulated cultures and in cultures exposed to TNP-LPS at any of the concentrations used (**Figure 1B**).

L-lysine supplementation did not significantly affect the percentage of IgM^+^ B cells in non-stimulated cultures or in cultures stimulated with TNP-LPS (**Figure 1C**).

### 3.2 Effect of L-methionine, L-tryptophan and L-lysine supplementation on proliferation of splenic IgM^+^ B cells

**Figure 2:**
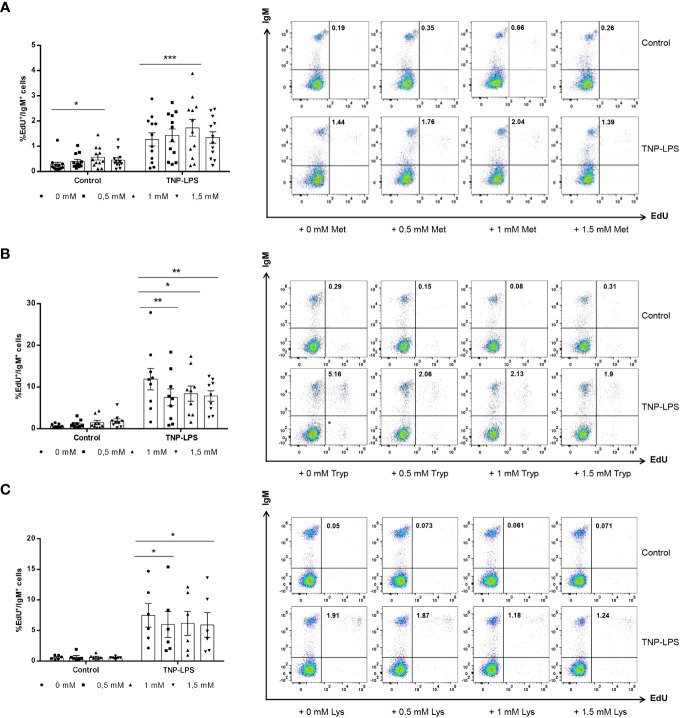
Effect of L-methionine, L-tryptophan and L-lysine supplementation on proliferation of IgM^+^ B cells. Spleen leukocytes were cultured in RPMI medium with 5% FCS and antibiotics supplemented with different concentrations of L-methionine **(A)**, L-tryptophan **(B)** or L-lysine **(C)** as described in the legend of **Figure 1**. Non-supplemented controls incubated with RPMI media alone were also included. Some of these cultures were additionally stimulated with 5 μg/ml of TNP-LPS, while others were left unstimulated. All cells were incubated for 3 days at 20°C. After this time, cells were labeled with EdU (1μM) and incubated for a further 24h. Thereafter, cells were labeled with anti-IgM mAb. The percentage of IgM^+^ B cells with incorporated EdU (proliferating IgM^+^ B cells) within the total IgM^+^ B cell population was calculated. Representative dot plots are included along with graphs showing the quantification of EdU^+^IgM^+^ cells among IgM^+^ B cells (mean + SEM) (n=12 for A, n=12 for B and n=6 for C). Symbols represent individual values. Asterisks denote significantly different values between cultures supplemented with amino acids and non-supplemented controls as indicated * means *p* ≤ 0.05, ** means *p* ≤ 0.01 and *** means *p* ≤ 0.001.

As described before (28), TNP-LPS induced a strong proliferation of IgM^+^ B cells in splenic leukocyte cultures. This TNP-LPS-induced IgM^+^ B cell proliferation was further elevated when 1 mM L-methionine was added to the cultures (**Figure 2A**). Surprisingly, even in the absence of TNP-LPS, L-methionine supplementation at 1 mM slightly increased the number of IgM^+^ B cells that were spontaneously proliferating (**Figure 2A**).

On the contrary, when leukocyte cultures were supplemented with L-tryptophan at any of the doses used, the proliferation of IgM^+^ B cells in response to TNP-LPS significantly decreased (**Figure 2B**). The supplementation of cultures with 0.5 mM or 1.5 mM L-lysine also provoked a significant down-modulation of the TNP-LPS induced proliferation (**Figure 2C**).

### 3.3 Effect of L-methionine, L-tryptophan and L-lysine supplementation on IgM secretion

**Figure 3:**
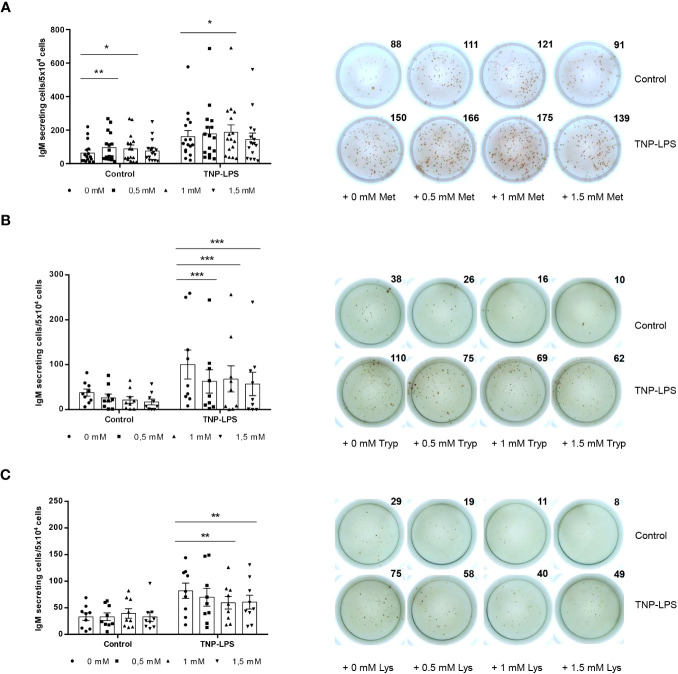
Effect of L-methionine, L-tryptophan and L-lysine supplementation on IgM secretion. Spleen leukocytes were cultured in RPMI medium with 5% FCS and antibiotics supplemented with different concentrations of L-methionine **(A)**, L-tryptophan **(B)** or L-lysine **(C)** as described in the legend of **Figure 1**. Non-supplemented controls incubated with RPMI media alone were also included. Some of these cultures were additionally stimulated with 5 μg/ml of TNP-LPS, while others were left unstimulated. All cells were incubated for 3 days at 20°C. Cells were plated in ELISpot plates previously coated with anti-IgM mAb (2 μg/ml) and incubated for a further 24 (h) After incubation, cells were washed away and a biotinylated anti-trout IgM mAb (1 μg/ml) used to detect number of spot forming cells. Duplicates for a representative individual are shown along with a quantification of spot forming cells from 16 **(A)**, 9 **(B)** and 9 **(C)** independent fish (mean + SEM). Symbols represent individual values. Asterisks denote significantly different values between cultures supplemented with amino acids and non-supplemented controls * means *p* ≤ 0.05, ** means *p* ≤ 0.01 and *** means *p* ≤ 0.001.

We performed an ELISpot to establish whether L-methionine, L-tryptophan and L-lysine supplementation could influence IgM secretion in splenocyte cultures. In the case of L-methionine, the 0.5 mM and the 1 mM concentrations increased the number of cells secreting IgM in leukocyte cultures (**Figure 3A**). In the presence of TNP-LPS, the number of IgM-secreting cells in cultures was strongly augmented as described before (28). This effect was further enhanced when cultures were supplemented with 1 mM L-methionine (**Figure 3A**).

In the case of L-tryptophan, the three doses tested significantly reduced the number of cells secreting IgM in cultures in TNP-LPS-stimulated cultures (**Figure 3B**). Finally, L-lysine supplementation at doses of 1 mM and 1.5 mM significantly down-regulated the number of IgM-secreting cells in the presence of TNP-LPS (**Figure 3C**).

### 3.4 Effect of L-methionine supplementation on the phagocytic capacity of splenic leukocytes

**Figure 4:**
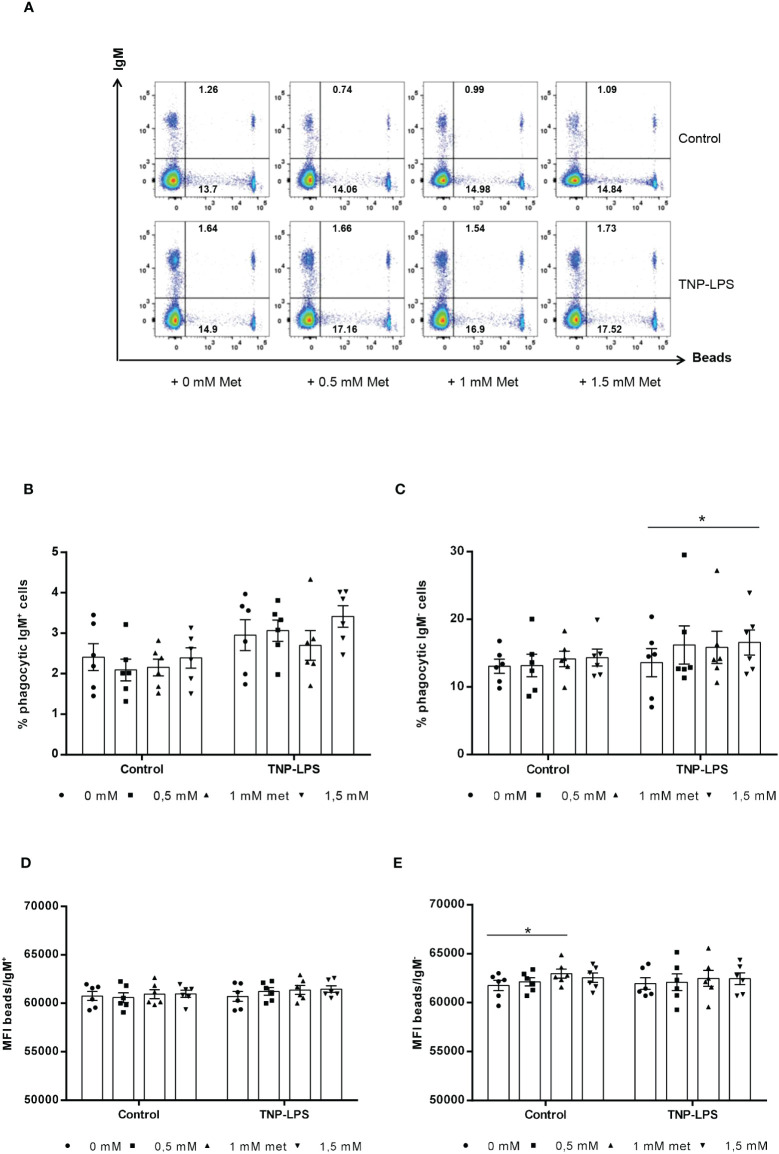
Effect of L-methionine supplementation on the phagocytic capacity of IgM^+^ B cells. Spleen leukocytes were cultured in RPMI medium with 5% FCS and antibiotics supplemented with different concentrations of L-methionine as described in the legend of **Figure 1**. Non-supplemented controls incubated with RPMI media alone were also included. Some of these cultures were additionally stimulated with 5 μg/ml of TNP-LPS, while others were left unstimulated. All cells were incubated for 3 days at 20°C. After this time, cells were incubated with Crimson Red fluorescent beads (1 μm diameter) at a ratio of 1:10 (cell/beads) for a further 3 h at 20°C. Non-ingested beads were removed by centrifugation over a cushion of 3% BSA supplemented with 4.5% D-glucose. Cells were then stained with anti-IgM mAb and analyzed by flow cytometry. **(A)** Representative dot plots for each experimental condition are shown. **(B, C)** Quantification of the percentage of phagocytic IgM^+^ B cells (cells in the upper right quadrant) among total IgM^+^ cells (cells in upper quadrants) **(B)** as well as a quantification of the percentage of phagocytic IgM^-^ B cells (cells in the lower right quadrant) among total IgM^-^ cells (cells in lower quadrants) are included **(C)**, along with the mean fluorescence intensity (MFI) of internalized beads within phagocytic IgM^+^ (upper right) **(D)** and IgM^-^ (lower right) cells **(E)** (mean + SEM; n = 6) (*p ≤ 0.05). Symbols represent individual values. Asterisks denote significantly different values between cultures supplemented with amino acids and non-supplemented controls (*p ≤ 0.05).

Since fish IgM^+^ B cells are known to have a potent phagocytic capacity (29), we also studied whether L-methionine supplementation could also modulate this activity. For this, splenocyte cultures were incubated in the presence of different L-methionine doses in presence or absence of TNP-LPS for 3 days. At that point, cells were exposed to fluorescent polystyrene beads for 3 h and the phagocytic capacity of IgM^+^ and IgM^-^ cells established by flow cytometry. In this case, L-methionine had no significant effects on the percentage of IgM^+^ B cells showing phagocytic activity (**Figures 4A, B**), nor on the phagocytic index, expressed as the mean fluorescence intensity (MFI) of beads within the phagocytic IgM^+^ B cell population (**Figures 4A, D**). Interestingly, the percentage of IgM^-^ cells with internalized beads significantly increased in cultures treated with TNP-LPS and supplemented with 1.5 mM L-methionine when compared to the percentage observed in cultures treated with TNP-LPS in which no additional L-methionine was added (**Figures 4A, C**). However, the phagocytic index was not affected in these conditions, and was only slightly increased in non-stimulated cultures exposed to 1 mM L-methionine (**Figures 4A, E**).

### 3.5 Effect of L-methionine supplementation on IgM secretion *in vivo*

**Figure 5:**
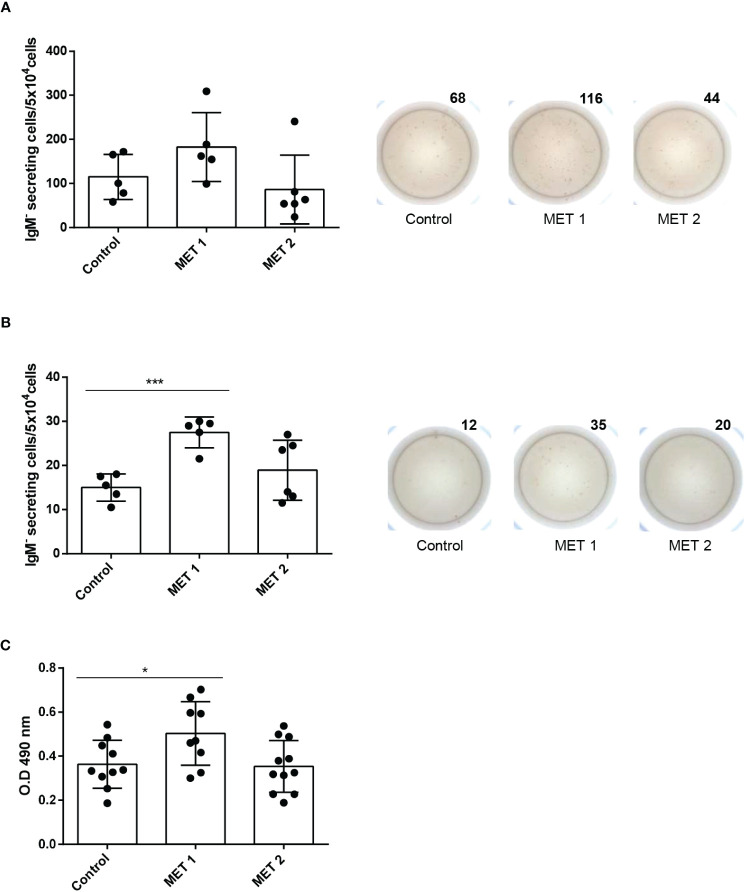
IgM B cell responses in fish fed diets containing different amounts of L-methionine. Rainbow trout were fed a control diet (CONTROL) or diets supplemented with methionine to make up for a 1.21% (MET 1) or a 1.58% methionine (MET 2) for 30 days. After that time, some fish were sacrificed and splenic **(A)** and blood **(B)** leukocytes isolated. Leukocytes were immediately plated in ELISpot plates previously coated with anti-IgM mAb (2 μg/ml) and incubated for 24 (h) After incubation, cells were washed away and a biotinylated anti-trout IgM mAb (1 μg/ml), used to detect number of spot forming cells. Wells obtained from a representative individual are shown along with a quantification of spot forming cells from 6 independent fish (mean + SEM). **(C)** At this point, other fish were anally immunized with TNP-LPS as described in Material and methods. After one month, fish were sacrificed and the TNP-specific IgM titers in serum detected by ELISA. Results are shown as mean values of absorbance at 490 nm. Dots represent individual values. Asterisks denote significantly different number of IgM-secreting cells in leukocytes obtained from fish fed a supplemented diet and fish fed the control diet (*p ≤ 0.05 and *** *p* ≤ 0.001).

We wanted to confirm the positive effects of methionine supplementation in a focused feeding experiment. For this, fish were fed either a control diet or diets containing a methionine surplus (MET 1 or MET 2). After 1 month of feeding, fish were sacrificed and used to obtain splenic and blood leukocytes and determine the number of IgM-secreting cells by means of ELISpot. Splenic leukocyte cultures from fish fed the MET 1 diet had a higher number of IgM-secreting cells, although the difference was not significant when compared to that of fish fed the control diet (**Figure 5A**). However, the effect was much more pronounced in the blood. Thus, blood leukocytes obtained from fish fed the MET 1 diet had a significantly higher number of IgM-secreting cells than fish fed the control diet (**Figure 5B**), thus confirming the positive effects of methionine supplementation on IgM secretion.

### 3.6 Effect of L-methionine supplementation on the specific response to anal immunization

To determine whether this positive effect that methionine supplementation was having on the IgM^+^ B cell response could translate into a more effective response to vaccination, we anally immunized fish with TNP-LPS following feeding for a month with either a control diet or diets containing a methionine surplus (MET 1 or MET 2), and then assessed the specific IgM response to the antigen. As shown in **Figure 5C**, the titers of TNP-specific IgMs were significantly higher in fish fed the MET 1 diet than those of fish fed the control diet. This difference was not visible in the fish fed the MET 2 diet (**Figure 5C**).

## 4 Discussion

Several recent studies have demonstrated the beneficial effects of methionine supplementation on different innate immune parameters of fish. For example, European seabass (*Dicentrarchus labrax*) fed diets enriched in methionine showed an increased percentage of neutrophils in the blood and a decreased expression of apoptotic genes after 12 weeks (30). Another study undertaken in European seabass corroborated these effects and demonstrated a higher survival of fish fed supplemented diets after a bacterial challenge, ultimately confirming the positive effects of methionine administration on the immune status (16). In rainbow trout, methionine surplus in the diet also increased the percentage of peripheral neutrophils while reducing the expression of pro-inflammatory genes (31). However, to date, the effect of methionine supplementation had never been studied on fish B cells. This relation between methionine and B cell function has been best explored in birds. Hence, methionine deficiency was shown to decrease plasma IgM and IgG levels in broilers (32) as well as IgA^+^B cells and the contents of IgA, IgG, and IgM in the intestine (33). In the current study, we have confirmed these effects of methionine on Ig secretion and B cell survival in fish, as methionine supplementation increased the number of cells secreting IgM and augmented the proliferative response of IgM^+^ B cells. Yet, it seems that not all B cell functions are equally affected by methionine, as the phagocytic capacity of trout B cells was not significantly modified in response to the presence of additional methionine in the medium.

Although the exact mechanisms by which methionine affects B cell function have not been addressed in any animal species to our knowledge, methionine is known to have different effects at cellular level that could ultimately impact B cell function. For example, methionine is required for protein synthesis (34), and B cells heavily rely on protein production during their differentiation process towards antibody secreting cells. Therefore, it seems obvious that methionine availability would influence the production of antibodies by affecting the overall protein synthesis capacity of B cells. Additionally, methionine is involved in DNA methylation, a process that regulates gene expression by adding methyl groups to DNA. Epigenetic modifications, including DNA methylation, play a significant role in B cell development and differentiation (35). Hence, it could also be possible that methionine availability may affect the epigenetic regulation of genes involved in B cell function, potentially influencing their response. The potent antioxidant activity of methionine might also affect the function of immune cells. Methionine is a precursor of glutathione, a potent antioxidant that protects cells from oxidative stress (36). B cells are exposed to oxidative damage during the immune response; therefore, maintaining an adequate supply of antioxidants is crucial for their optimal functioning (37). Finally, methionine is a key component of the one-carbon metabolism pathway, which is involved in various cellular processes, including nucleotide synthesis and methylation reactions (34). B cells are known to require nucleotides for their proliferation and antibody production (38). Therefore, methionine availability can influence the availability of one-carbon units required for nucleotide synthesis, thereby affecting B cell proliferation and antibody production.

The positive effects provoked by methionine surplus in the medium on trout splenic IgM^+^ B cells were not similarly exerted by tryptophan and lysine, which in some cases even provoked negative effects on B cell survival, proliferation and IgM secretion. Thus, tryptophan and lysine appear to be harmful to trout IgM^+^ B cells at least at the concentrations and incubation times tested in the present study. In contrast with our results, Bonezi and colleagues (39) reported a correlation between tryptophan metabolism and the differentiation of B cells to antibody-secreting cells observed in patients infected with Dengue virus. Interestingly, this connection was specific of Dengue virus and other flaviviruses with lesser capacity to induce a strong production of antibody-secreting cells also induced a lower tryptophan catabolism in culture (39). In fish, the effects of tryptophan have been mostly studied in relation to stress. Thus, for example, while tryptophan supplementation of Senegalese sole (*Solea senegalensis*) in the diet had higher mortality in response to a bacterial infection, it had positive effects on fish kept at high densities, and was therefore suggested as a promising strategy to overcome chronic stress-induced disease susceptibility in farmed fish (22). Therefore, it seems possible, that the effects that tryptophan supplementation might have on B cells *in vivo* will also be dependent on the levels of cortisol and the overall physiological status of the fish, as well as on the bi-directional communication of neuro-endocrine and immune systems. Thus, further research is required to understand the direct effects of tryptophan availability on B cells and how tryptophan can affect the immune response of these and other immune cells in fish. The effect that lysine supplementation has on the immune response of fish has been scarcely studied to date. In grass carp (*Ctenopharyngodon idellus*), significantly increased the transcription of cytokines such as transforming growth factor β2 (TGFβ2) and interleukin 4/13B (IL4/13B) in the intestine (40), yet the implications that these effects may have on immune cells were not investigated.

The positive effects of methionine supplementation on IgM secretion were further confirmed in a feeding trial, in which fish were fed either a control diet or diets supplemented with two different amounts of methionine. One of this supplemented diets (MET 1 containing 1.21% methionine), provoked a significant up-regulation of the number of IgM-secreting cells in blood, thus confirming the results obtained in the *in vitro* trial. Finally, to establish whether these beneficial effects on B cell functionality translated into a higher IgM^+^ B cell response to an antigen, we anally immunized fish with TNP-LPS and evaluated the specific IgM response after 1 month. This immunization protocol had been previously demonstrated to induce a robust specific IgM response at systemic level (27). Remarkably, what we found was that again the group of fish fed the MET 1 mounted a significantly higher TNP-specific IgM response than those fed with the control diet. Again, the response to the MET 2 diet was not significantly different from that of fish fed the control diet, suggesting that methionine surplus has beneficial effects up to a certain level and, after that point, the positive effects can be reverted. This could be a result of a negative feedback mechanism turned on in response to excessive methionine availability, yet this is something that needs to be further investigated.

In conclusion, while tryptophan and lysine surplus presented clear negative effects on B cells, methionine supplementation seemed to be highly beneficial for the functionality of trout IgM^+^ B cells. This was demonstrated in a series of *in vitro* studies using splenic leukocyte cultures. Methionine supplementation of these leukocyte cultures specifically up-regulated the survival, proliferation and IgM secreting capacities of IgM^+^ B cells, while it had no effects on their phagocytic activity. *In vivo*, methionine supplementation provoked an up-regulation of the number of cells secreting IgM in blood. Importantly, we also found that fish fed the diet containing 1.21% methionine (MET 1) were able to mount a significantly higher specific IgM response, results with important practical implications for aquaculture. The data obtained provide new insights on the role of methionine on fish B cells, and demonstrate for the first time positive effects of dietary methionine supplementation on the adaptive immune response of fish.

## Data availability statement

The raw data supporting the conclusions of this article will be made available by the authors, without undue reservation.

## Ethics statement

The animal study was approved by INIA. Consejo Superior de Investigaciones Científicas (CSIC). The study was conducted in accordance with the local legislation and institutional requirements.

## Author contributions

CT: Conceptualization, Funding acquisition, Supervision, Writing – original draft. DM: Data curation, Formal Analysis, Investigation, Methodology, Writing – review & editing. MO: Investigation, Methodology, Writing – review & editing. IC: Investigation, Methodology, Writing – review & editing. PD: Formal Analysis, Investigation, Methodology, Supervision, Writing – review & editing. NN: Investigation, Methodology, Writing – review & editing. SV: Investigation, Methodology, Writing – review & editing. AA: Investigation, Methodology, Writing – review & editing. CZ: Conceptualization, Writing – review & editing. MM: Formal Analysis, Writing – review & editing. BC: Conceptualization, Supervision, Writing – review & editing.
